# Supplementary material for: Surface Properties of Parabacteroides distasonis and Impacts of Stress-Induced Molecules on Its Surface Adhesion and Biofilm Formation Capacities
Source: Microorganisms. 2021 Jul 27;9(8):1602. doi: 10.3390/microorganisms9081602 (PMC8400631; doi:10.3390/microorganisms9081602)
Supplement: Supplementary file 1 [file microorganisms-09-01602-s001.zip › microorganisms-1287789-supplementary.pdf]

Surface properties of *Parabacteroides distasonis* and impacts of stress-induced molecules on its surface adhesion and biofilm formation capacities

Jordan Chamarande [1], Lisiane Cunat [1], Céline Caillet [2], Laurence Mathieu [3], Jérôme F.L. Duval [2], Alain Lozniewski [1,4], Jean-Pol Frippiat [1], Corentine Alauzet [1,4] and Catherine Cailliez-Grimal [1,*]

Supplementary material

Molecular typing of P. distasonis strains.

In order to assess that strain diversity was well represented within the species *P. distasonis*, epidemiological link between 19 clinical strains isolated at the microbiology laboratory of the University Hospital of Nancy, France, and the type strain *P. distasonis* DSM 20701^T has been investigated. Clonality was assessed using Arbitrarily Primed-PCR (AP-PCR, primer sequence: 5′-GGTTGGGTGAGAATTGCACG-3′) and Enterobacterial Repetitive Intergenic-PCR (ERIC-PCR, primer sequence: 5′-AAGTAAGTGACTGGGGTGAGCG-3′) as previously described **[31,32]**. The extracted DNA was added to 50 µL of PCR mixture containing dNTPs, $MgCl_2$ (50mM), PCR buffer (10X), Taq polymerase (5u/µL) and ERIC or AP primer (50 µM). The DNA was amplified by the MyiQTM Two-Color (BioRad) device using the following program: 94 °C for 5 minutes, 94 ° C for 1 minute, 25 °C for 1 minute, 72 °C for 2 minutes, 40 cycles. The amplicons were then separated and revealed by gel electrophoresis (50V, 1 hour 35 minutes). The DNA fingerprints of the 20 strains obtained by both methods **Figure S1**) were visually compared by two observers (LC and CA) and interpreted according to the following criteria: two strains were assigned to distinct clonal group if their patterns differed by more than one major band or two minor bands by at least one method.

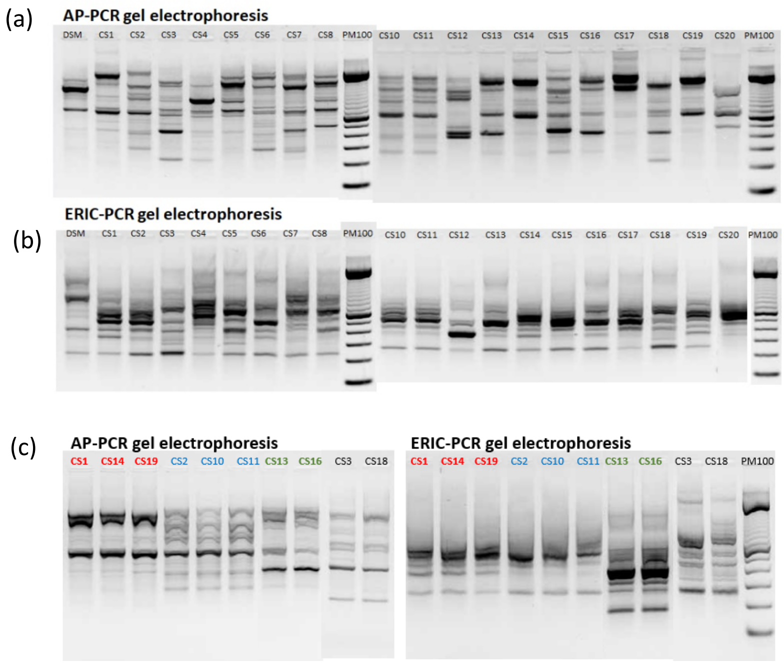

Figure S1. Arbitrarily Primed-PCR (a) and Enterobacterial Repetitive Intergenic-PCR (b) fingerprinting patterns of 20 strains of *P. distasonis*. Identical patterns revealed by visual observation (c) allowed the elimination of 6 strains and the selection of the 14 non-clonal strains: DSM, CS1 (clonal to CS14 and CS19, these two latter being eliminated), CS2 (clonal to CS13 and CS11, these two latter being

eliminated), CS4, CS5, CS6, CS7, CS8, CS12, CS13 (clonal to CS16, this latter being eliminated), CS15, CS17, CS18 (clonal to CS3, this latter being eliminated) and CS20.

Table S1. Survivability of *P. distasonis* DSM 20701^T under experimental conditions (CFU/mL).

	T_{1h}	T_{2h}	T_{4h}	T_{6h}	T_{24h}	T_{48h}
Saline solution RT, A		2,0E+08 +/- 1,7E+07	1,8E+08 +/- 4,4E+06	2,1E+08 +/- 1,1E+07	ND	ND
Diluted MH RT, An		1,9E+08 +/- 3,6E+06	2,0E+08 +/- 5,7E+06	2,0E+08 +/- 2,9E+07	1,9E+08 +/- 1,0E+07	1,8E+08 +/- 5,5E+07
$NaNO_3$ 1 mM RT, A	2,6E+08 +/- 4,4E+07					
$NaNO_3$ 250 mM RT, A	2,3E+08 +/- 3,7E+07					

RT: room temperature, A: aerobic conditions, An: anaerobic conditions, +/-: standard deviation, ND: Not detectable. Mediums were inoculated with 10^8 CFU/mL (T0h). Enumerations were realized after 2, 4, 6, 24 and 48 hours in saline solution and diluted MH broth and after 1 hour in 1 and 250 mM $NaNO_3$ solution.

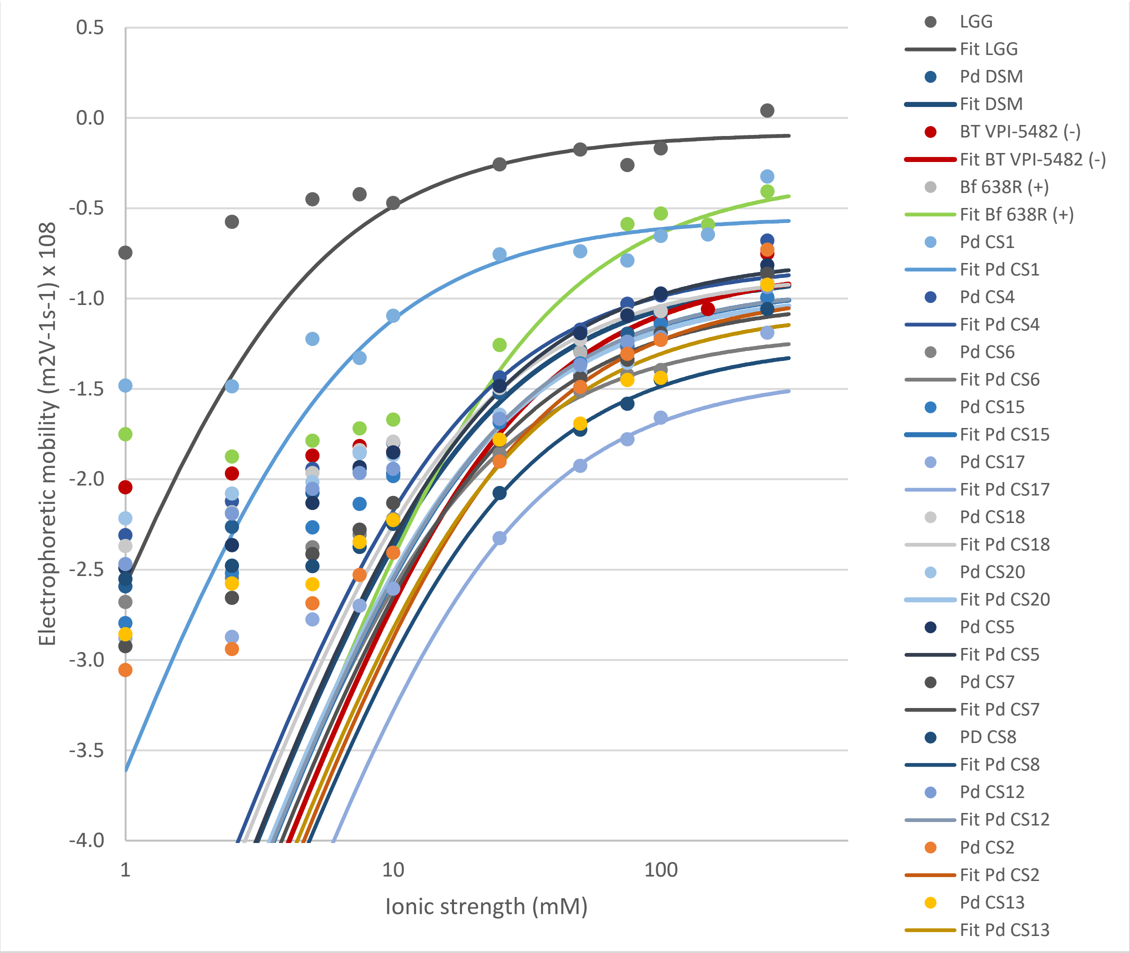

Figure S2. Impact of electrolyte concentration on the electrophoretic mobility of 14 *P. distasonis* strains and controls LGG, *Bf* 638R and *Bt* VPI-5482.
